# Detective flow imaging, directional enhanced blood flow imaging, and contrast-enhanced harmonic endoscopic ultrasound in pancreatic solid lesions

**DOI:** 10.1055/a-2590-8391

**Published:** 2025-05-22

**Authors:** Filippo Antonini, Durante Donnarumma, Tiziana Buono

**Affiliations:** 1 220399 Department of Gastroenterology and Interventional Endoscopy, Mazzoni Hospital AST Ascoli Piceno, Ascoli Piceno, Italy

Contrast-enhanced harmonic endoscopic ultrasound (CH-EUS) is known for its superior diagnostic accuracy for solid pancreatic lesions (SPLs), primarily due to its enhanced visualization of perfusion patterns 1 . However, it may not be suitable for all patients and relies on the use of expensive contrast agents. In contrast, directional enhanced blood flow imaging (eFLOW) and the more recent detective flow imaging endoscopic ultrasound (DFI-EUS) are innovative imaging techniques that improve the visualization of fine vascular structures in various tissues, all without the need for contrast agents 2 3 .

We present a brief series of cases of both malignant and benign SPLs evaluated through eFLOW and DFI-EUS, with a comparative analysis using CH-EUS ( Fig. 1 , Fig. 2 , Video 1 ). Notably, this series includes a rare case highlighting the application of DFI in a patient diagnosed with autoimmune pancreatitis.

**Fig. 1:**
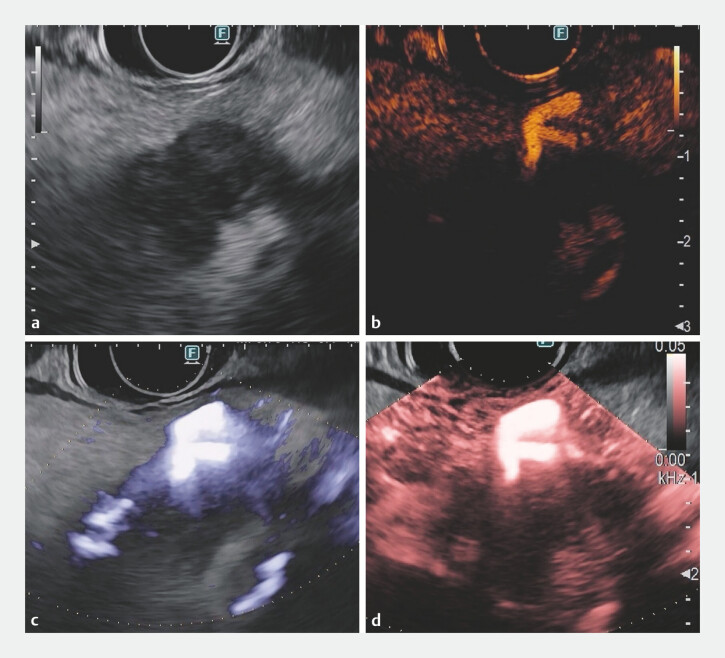
A case of pancreatic cancer: **a** B-mode imaging reveals a hypoechoic lesion. On ( **b** ) CH, ( **c** ) directional eFLOW, and ( **d** ) DFI endoscopic ultrasound, the tumor consistently shows a hypovascular pattern with central areas of increased blood flow. Abbreviations: CH, contrast-enhanced harmonic; DFI, detective flow imaging; eFLOW, enhanced blood flow imaging.

**Fig. 2:**
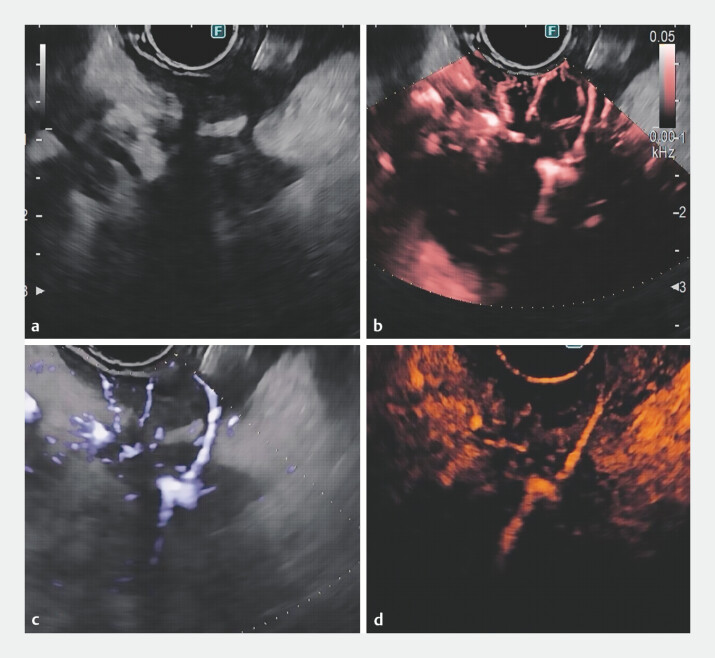
A case of degenerated IPMN: **a** B-mode imaging shows a hypoechoic area with irregular margins. On ( **b** ) DFI, ( **c** ) directional eFLOW, and ( **d** ) CH endoscopic ultrasound, the characteristic hypo-enhancement of pancreatic cancer is observed, along with a linear central vessel. A diagnostic EUS-guided fine needle biopsy was performed on the hypoechoic area, carefully avoiding the vessel. Abbreviations: CH, contrast-enhanced harmonic; DFI, detective flow imaging; eFLOW, enhanced blood flow imaging; EUS, endoscopic ultrasound; IPMN, intraductal papillary mucinous neoplasm.

Solid pancreatic lesions evaluated through directional enhanced blood flow imaging (eFLOW) and detective flow imaging (DFI), with a comparative analysis using contrast-enhanced harmonic endoscopic ultrasound (CH-EUS).Video 1

CH-EUS, DFI-EUS, and eFLOW can provide complementary insights into the vascular characteristics of the lesions. All three techniques tend to reveal similar findings in terms of vascular patterns: in pancreatic adenocarcinoma, these imaging modalities typically show a hypovascular pattern, reflecting the poor blood supply and disorganized blood vessels that are characteristic of malignancy, often associated with areas of increased blood flow or neoangiogenesis within the tumor.

While CH-EUS typically offers the most comprehensive analysis of blood perfusion in SPLs, established criteria for classifying vessels with DFI-EUS and eFLOW are still lacking. However, using these modalities in a complementary manner can significantly enhance the differential diagnosis of pancreatic lesions. Both eFLOW and DFI-EUS provide valuable alternatives, especially for patients who are unable to receive contrast agents.

Endoscopy_UCTN_Code_TTT_1AS_2AD

## Notes

Endoscopy E-Videos https://eref.thieme.de/e-videos
*E-Videos* is an open access online section of the journal *Endoscopy* , reporting on interesting cases and new techniques in gastroenterological endoscopy. All papers include a high-quality video and are published with a Creative Commons CC-BY license. Endoscopy E-Videos qualify for HINARI discounts and waivers and eligibility is automatically checked during the submission process. We grant 100% waivers to articles whose corresponding authors are based in Group A countries and 50% waivers to those who are based in Group B countries as classified by Research4Life (see: https://www.research4life.org/access/eligibility/ ). This section has its own submission website at https://mc.manuscriptcentral.com/e-videos .
